# Targeting Divergent Pathways in the Nutritional Management of Depression

**DOI:** 10.3390/nu16162806

**Published:** 2024-08-22

**Authors:** Derek Tobin, Alexander Vuckovic, Jerome Sarris

**Affiliations:** 1 Tobin AS, 1352 Kolsås, Norway; 2 McLean Hospital, Belmont, MA 02478, USA; 3 NICM Health Research Institute, Western Sydney University, Westmead, NSW 2145, Australia; 4 The Florey Institute of Neuroscience and Mental Health, Melbourne University, Melbourne, VIC 3052, Australia; 5 Centre for Mental Health, Swinburne University of Technology, Melbourne, VIC 3122, Australia

**Keywords:** depression, nutrition, omega-3, EPA, methylfolate, s-adenosylmethionine, SAMe

## Abstract

The nutritional management of depression has long been discussed, due to the perceived benefit of a nutritional product having less side effects than pharmaceutical agents. Candidate nutrients for managing depression include vitamin D, B vitamins, tryptophan, branch chain amino acids, probiotics, omega-3 fatty acids, folate/methylfolate (also known as vitamin B9), and s-adenosylmethionine. This paper provides a narrative review of three nutrients which have significant scientific support for the management of depression. A deficiency in each nutrient is associated with depression, and interventional studies indicate that the correction of the nutritional deficiency may provide clinical benefit. We present epidemiological evidence, a mechanistic explanation and a review of interventional studies for these nutrients. Finally, relevant nutritional guidelines are presented with their conclusion for the role of each nutrient in the management of depression.

## 1. Introduction

Major Depressive Disorder (MDD) is a debilitating condition which constitutes one of the most prevalent mental health diagnoses globally. MDD induces profound impairments in cognitive, affective, and sensory experiences [1]. The World Health Organization (WHO) estimates that approximately 322 million people are currently affected by MDD (inclusive of dysthymia, now termed Persistent Depressive Disorder) [2]. This represents an increase exceeding 18% within the past decade, solidifying MDD’s position as the leading global cause of disability and ill health [2].

It is worth noting that first-line antidepressants, typically selective serotonin re-uptake inhibitors (SSRIs) or serotonin-norepinephrine re-uptake inhibitors (SNRIs), fail to achieve remission in approximately two-thirds of patients with depression [3]. In addition, SSRI/SNRIs have side effects, such as dietary disorders, reduced sexual function, headaches, insomnia, dizziness, sweating, tremors and anxiety. The poor efficacy often experienced with first-line medications may be partly due to the fact that depression arises from a complex interplay of factors. Effective treatments may therefore require a multi-component approach in order to target these diverse mechanisms that result in depression [4].

One potential approach to managing depression, as either monotherapy or as an adjunct to antidepressants, involves the use of nutritional supplementation, which can include vitamins, minerals, amino acids, phytonutrients, fatty acids, and other nutrient-based compounds. While the efficacy of many nutrient supplements for treating MDD is questionable, several have garnered considerable support from convergent sources of evidence, from preclinical (mechanistic) research to randomised controlled trials.

For instance, Howland (2012) performed a review of methylfolate, S-adenosyl-L-methionine (SAMe), tryptophan, omega-3 fatty acids and inositol, and their role in the management of depression [5]. Of these five nutrients, only omega-3 fatty acids and SAMe were shown to have sufficient substantiation to warrant their safe use. A similar nutraceutical review was performed by Lande in 2020 [6], with the conclusion that there was sufficient scientific evidence for the use of SAMe, methylfolate, omega-3 fatty acids, and vitamin D for the management of depression.

An additional review of the clinical trials of nutritional supplements for MDD [7] found support for the use of omega-3, methylfolate, SAMe, and vitamin D, as adjuncts to antidepressants for a reduction in depressive symptoms. Subsequently, a broad umbrella review conducted in 2019, which aggregated data from 33 meta-analyses of placebo-randomised controlled trials (RCTs) across 10,951 individuals with psychiatric disorders, found that the strongest evidence across the nutritional landscape was for eicosapentaenoic acid (EPA) as an adjunctive treatment for depression. This was closely followed by the positive data from RCTs of high-dose methylfolate in major depressive disorder, whilst other nutraceuticals (such as vitamin D, antioxidants and probiotics) had mixed or inconsistent results [8].

Such evidence has also been incorporated and summarised within global clinical guidelines for nutrient-based treatments of mental disorders, with the World Federation of Societies of Biological Psychiatry (WFSBP) guidelines supporting omega-3 and L-methylfolate usage for depression, with SAMe also having potential therapeutic use, while noting limitations with lower doses or poorer quality formulas [9].

The current paper provides a narrative review for three nutrients that have significant scientific support for a role in depression. Whilst other nutrients may also be important, this narrative focuses on omega-3, methylfolate and S-adenosylmethionine. The various mechanisms and pathways these nutrients act through are described, with their efficacy demonstrated by interventional studies. Thus, the article provides a snapshot of our current understanding of three important nutrient adjuncts for depression management.

## 2. Materials and Methods

A review of the literature relating to the nutritional components omega-3 lipids, methyl-folate and SAMe was performed. Searches were performed for the terms «depression» and «omega-3». Omega-3 observational studies were retrieved from the GOED clinical database [10] and PUBMED with the search “omega-3” “depression” from 2005 onwards. Omega-3 interventional studies were retrieved from the GOED clinical database and PUBMED, reported from 2016 to 2024, using the terms “omega-3” AND “depression” and limited to studies after 2016. The search was performed in April 2024.

For the retrieval of studies on methylfolate, the term “folate” was used for epidemiological studies and/or “methyl-folate” for interventional studies).

The studies reported for s-adenosylmethionine were retrieved from PUBMED with the terms “S adenosylmethionine” and “Depression” after 1990. The search was performed in July 2024. Studies reporting depression in Parkinson’s patients and patients with HIV/AIDS were excluded.

The relevant studies needed to be in English, with an available abstract. The studies were only selected if they referred to “major depressive disorder”, “depression”, depressive disorders” or pregnancy-related depression (e.g., post-partum depression). Studies were excluded if they reported only psychosis, bipolar depression, or other mental disorders other than depression.

Interventional studies for all nutrients were selected with the PUBMED limiting criteria “clinical trial” or “randomized controlled trial”.

## 3. Results

### 3.1. Omega-3 Fatty Acids

#### 3.1.1. Review of Mechanistic Data

EPA is considered to have an important role in the maintenance of mood (e.g., depression) due to its role as an anti-inflammatory agent with neurochemical modulatory properties [11]. This hypothesis is supported by the findings of Firth et al., who reports in a meta-analysis of 14 interventional trials that benefits of EPA were noted in subjects with systemic inflammation and not in those with depression related to illness (i.e., non-inflammatory) [8]. In addition, higher doses of EPA had the greatest effect on depression and, interestingly, a minimal ratio of EPA:DHA of 2:1 was demonstrated for optimal efficacy.

#### 3.1.2. Neurochemical Modulation

EPA may also affect neuronal function. Synaptic plasticity increases with EPA, which may play a role in neurotransmitter release and uptake and in ion channel activity, considered essential components of depression [12]. Apoptosis from injury is reduced and EPA stimulates brain-derived neurotrophic factor (BDNF) production, which plays a role in neuronal function and health. The plasma membrane incorporation of EPA not only influences membrane fluidity, but also competes with membrane-bound arachidonic acid (AA). This is particularly true for EPA which, with a similar carbon length to AA, competes with metabolising enzymes. AA is the precursor for inflammatory signalling molecules, and EPA competition reduces the levels of these inflammatory prostaglandins, leukotrienes, and thromboxane [13]. In addition, EPA incorporation into mitochondrial membranes stabilises mitochondrial function and reduces oxidative stress, a major factor for neuronal health, particularly in Alzheimer’s Disease [14].

#### 3.1.3. Specialised Pro-Resolving Mediators

In 2000, Serhan et al. reported that inflammation resolution was a biochemically controlled active process, orchestrated by a host of lipid mediators called Specialised Pro-resolving Mediators (SPMs). This umbrella term includes families with distinct biological function and biosynthetic pathways, termed lipoxins, resolvins, protectins and maresins. The SPMs down-regulate the gene expression of inflammatory cytokines, reduce polymorphonuclear neutrophil (PMN) infiltration, decrease pro-inflammatory mediator production (both lipid mediators and cytokines), and stimulate the phagocytosis of apoptotic neutrophils by macrophages [15]. EPA, as well as DHA, DPA (docosapentaenoic acid) and arachidonic acid (AA), are precursors to a host of SPMs. The rapid metabolism of EPA to these pro-resolving mediators has been proposed as a hypothesis explaining why EPA is barely detectable in the brain. An association of SPMs, inflammation with depression has been recently reported in MDD [16].

#### 3.1.4. Endocannabinoids

The endocannabinoid system (ECS) is a neural system involved in the regulation of homeostasis. Cannabinoid receptors (CB1 and CB2) are found throughout the central and peripheral nervous system and play roles in diverse functions, such as satiety, energy balance, neuronal protection and the prevention of neurodegenerative diseases, with effects in stroke, mood disorders, emesis, pain, and inflammation. The natural ligands are lipid-derived endocannabinoids (eCBs), which are membrane-bound long chain fatty acids that are released upon neuronal activation. The two principal eCBs, Anandamide (ANA) and 2-AG, are AA-derived metabolites, while docosahexaenoyl ethanolamide is derived from DHA and oleylethanolamide, palmitoylethanolamide and EPA-ethanolamide are derived from EPA [17].

In a clinical trial with MDD patients given either EPA, EPA+DHA or DHA alone, supplementation led to increases in eCBs relevant to the type of supplementation. A significant benefit in the form of increased clinical remission was seen in those receiving EPA or EPA+DHA compared to DHA alone [18]. This endorses the role of EPA and its metabolite EPEA in modulating depression.

#### 3.1.5. Observational Studies of Omega-3 in Depression

Epidemiological studies are a means of showing population effects. This is of particular use in nutritional research, where nutritional effects are often long term, mild, and subject to influence by co-variants. Table 1 summarises the epidemiological data for omega-3 and depression. Many of the studies measure omega-3 fatty acids in blood. This is a more accurate means of measuring the dietary intake of omega-3 fatty acids than monitoring the intake of food. Measurements made in plasma are reflective of an acute intake (i.e., values vary according to daily intake), whereas red blood cell measurements reflect the overall intake over several months. The majority of epidemiological studies support an association between omega-3 intake with depression/mood changes.

The observational studies reported in Table 1 strongly support the association of dietary omega-3 with depression. Of the 13 studies reported above, 1 reported no association of mental health with omega-3 intake, 4 were equivocal and 8 reported an association of omega-3 intake with mental health.

A meta-analysis performed with 14 studies by Lin [32] showed that EPA and DHA, as well as total *n*-3 polyunsaturated fatty acids, were significantly lower in depressive patients compared to those without depression. No such association was found for arachidonic acid or total *n*-6 PUFA.

The above data provide two important points, firstly the condition of MDD in some people may create a particular nutrient requirement for anti-inflammatory nutrients, such as EPA. Secondly, there are compelling data that MDD patients have lower than normal levels of EPA either due to poor intake, or an increased metabolism of EPA.

The 2015–2020 Dietary Guidelines for Americans (DGA) recommend levels of seafood intake of approximately 250 mg of EPA and DHA daily [33]. An analysis of actual intakes using the NHANES database shows that women of childbearing age have less than half the DGA recommended intake [34]. NHANES data in women of child-bearing age show that supplementation provides an additional 26 mg, 62 mg and 88 mg of EPA, DHA and EPA+DHA, respectively. Consequently, over 95% of the study population did not have the recommended intake of 250 mg EPA and DHA daily.

#### 3.1.6. Interventional Studies of Omega-3 in Depression

A total of 151 intervention studies with omega-3 in the field of depression were reported for omega-3 and depression. A review of the studies reported from 2016 to 2024 is detailed below in Table 2.

Of the 21 studies listed, 17 studies reported improvements in depression with omega-3 supplementation. Of particular interest is the study by Parletta et al. Although this study did not specifically supplement with omega-3 (the study looked at the effect of a Mediterranean diet), depression was shown to be associated with red blood cell omega-3 levels, implying that an increase, or correction, of omega-3 levels is important in the normalisation of mood. Jang et al. confirmed this, showing that supplementation resulted in increased omega-3 blood levels and an improved mood; this study, and others showing dose dependency, support the concept that supplementation may potentially fulfil a nutritional requirement of EPA for the management of depression.

#### 3.1.7. Guidelines Relevant to Omega-3 Use in Managing Depression

The International Society for Nutritional Psychiatry Research has developed a 2019 guideline with regard to the use of omega-3 fatty acids in depression. The society guideline considers an effective dose of omega-3 for clinical use in MDD of 1–2 g of EPA daily. Interestingly, the Society also recommends that the ratio of EPA:DHA does not fall below 2:1 [56].

Clinical Psychopharmacology and Neuroscience published a clinical guideline in 2020 for children and adolescents with MDD, with the recommendation of a 1–2 g dose of EPA+DHA for 12–16 weeks. Again, the recommended ratio of EPA: DHA is at least 2:1, implying that EPA is the functional fatty acid [57].

Additionally, guidelines written to aid clinicians in the treatment of psychiatric disorders with nutrients have been developed by The World Federation of Societies of Biological Psychiatry (WFSBP) and Canadian Network for Mood and Anxiety Treatments (CANMAT) Taskforce [9]. The guideline recommends the use of 1 g to 2 g of EPA for adjunctive use in MDD but not as monotherapy.

This position was held due to EPA having strong clinical trial evidence for co-use with antidepressants; however, some more recent data from monotherapy studies are less compelling, and the weaker effect size may be due to people being included in studies with pre-existing normal or high fish consumption.

### 3.2. Folate and Methylfolate

#### 3.2.1. The Physiological Role of Folate/L-Methylfolate

Folate and its downstream metabolite, L-methylfolate, have pleiotropic activity, with a role in 1 carbon metabolism as an anti-inflammatory agent and as an important component in neurotransmitter synthesis [58]. Folate/methylfolate metabolism is important in the metabolism of homocysteine to methionine and thereafter to SAMe, which, amongst others, acts as a precursor to neurotransmitters. Increased homocysteine levels are associated with several chronic illnesses, including cardiovascular disease and dementia. The imbalance of homocysteine is also linked with neurotransmitter dysregulation [59].

#### 3.2.2. Anti-Inflammatory Role

In the case of mood disorders, methylfolate (the bioactive form of folate) is important in the methylation of homocysteine to methionine and thereafter in the production of S-adenosylmethionine (SAMe). Supplementation with 800 micrograms of folate over a 2-week period significantly raised plasma folate levels and importantly, significantly reduced circulating homocysteine levels [60]. Folate/methylfolate, therefore, has a role in the homeostatic control of homocysteine, which indirectly regulates neuroinflammation [61] related to mental health [59,62].

#### 3.2.3. One-Carbon Metabolism

The one-carbon cycle/folate metabolic pathway has widespread actions throughout the body, including the regulation of both nucleotide synthesis and DNA methylation [63]. DNA methylation is a form of epigenetic control of DNA expression that has an impact throughout the genome and affects all body systems.

The synthesis of the depression-associated neurotransmitters serotonin, dopamine, and norepinephrine is regulated by L-methylfolate. For a review of L-methylfolate activity, see Stahl, 2008 [64].

#### 3.2.4. Methylfolate Metabolism

L-methylfolate is the metabolic product of folate metabolism catalysed by the enzyme methylenetetrahydrofolate reductase (MTHFR). The enzyme converts 5,10-MTHF to 5-MTHF. 5-MTHF then donates a methyl group in the conversion of homocysteine to methionine [65].

Polymorphisms are genetic variations seen in a healthy population, and contribute to inter-individual variation. Several polymorphisms have been identified for the gene coding the folate metabolising enzyme MTHFR, resulting in reduced enzymatic activity with subsequent lower levels of L-methylfolate. Since only L-methlyfolate can cross the blood–brain barrier, the concentration of folate in any form in the brain is dependent on the circulating levels of L-methylfolate [66]. A decreased or absent expression of the MTHFR protein leads to decreased levels of 5-MTHF, which then leads to high levels of homocysteine. This results in the suboptimal production of monoamines, including serotonin, dopamine, and nor-epinephrine [67].

Polymorphisms in the MTHFR gene are seen in 2–20% of the general population, with frequency being dependent on ethnicity [68]. However, the prevalence of these polymorphisms appears to be 36–82% higher in people with severe mental illness, with meta-analyses of population data showing MTHFR polymorphisms associated with major depression (odds ratio (OR) 1.36), schizophrenia (OR 1.44) and bipolar disorder (OR 1.82) [68].

Polymorphisms in the MTHFR gene have been associated with depression. The Hordaland study demonstrated an association of the *C677T MTHFR* and hyperhomocysteinaemia with depression with an odds ratio of 1.69 [69]. In the same study, the frequency of depression was compared between different variants of the 677 nucleotide. Of the 240 women recruited, approximately half had the C to T variant. A study in a population from Northern Ireland also found significantly higher occurrence of the C677T polymorphism in those with a history of depression [70].

In a separate analysis of the British Women’s Heart and Health Study, a total of 3487 women were analysed for their MTHFR genotype. The C to T heterozygous genotype was seen in 43.6% of women [71], and these women had an associated increased risk of diagnosed depression. This finding is supported by a meta-analysis by the same authors of 8 smaller studies, with a total of 669 cases which demonstrated the association of C to T transition, with depression at an odds ratio of 1.36 [71].

Interestingly, a study of pregnant women showed that folate supplementation could protect against depression outside of pregnancy. However, the protective function of supplemental folate was lost in women with a C to T polymorphism, indicating that folate supplementation is not effective in women with a C to T polymorphism, as folate is not efficiently converted to methylfolate and therefore not carried across the blood–brain barrier [72].

#### 3.2.5. Observational Studies of Folate in Depression

Studies describing folate levels in psychiatric disorders are presented in Table 3.

The studies described above have varying designs in a variety of populations. Seven of the studies demonstrate an association between folate levels and depression, two studies showed no association and two studies showed gender specific outcomes. From the above studies, it would therefore appear that folate deficiency is seen in the MDD population. One study reported low folate levels in Latino women with MDD but no association in men. Further studies would be required to confirm whether depression and folate levels vary in different populations.

In conclusion, the majority of studies support that low folate levels are associated with a number of psychiatric disorders, including depression [82,83].

#### 3.2.6. Interventional Studies of Methylfolate in Depression

Intervention studies of supplemental methylfolate in depression are presented in Table 4.

Of the 12 intervention studies reported above, 10 showed significant improvements in MDD measurements, whilst 2 showed no effect of supplementation.

The intervention study by Godfrey et al. (1990) describes folate deficiency in 33% of depressives and that supplementation with methylfolate was beneficial for depression, implying that correction of the folate deficiency was instrumental in the management of depression [90]. This is further supported by the meta-analysis of MTHFR genotypes in depression; of particular interest in this analysis is the finding that folate concentrations were not associated with depression, but the incidences of MTHFR polymorphisms were [70]. Taken together, this presents two concepts, first that folate levels are associated with depression, and second that a subset of subjects with MTHFR polymorphisms may have a folate-independent reduction in L-methylfolate.

#### 3.2.7. Guidelines Relevant to Methylfolate Use in Managing Depression

The World Federation of Societies of Biological Psychiatry (WFSBP) and Canadian Network for Mood and Anxiety Treatments (CANMAT) Taskforce is a publication providing guidelines on nutrient use for depression. The guideline recommends 15 mg per day of methylfolate for adjunctive use in MDD. Interestingly, they do not recommend folic acid [9]. This position was held due to the majority of the evidence being for methylfolate, with certain larger studies involving folic acid not demonstrating antidepressant effects.

### 3.3. S-Adenosyl Methionine

#### 3.3.1. Mechanism of Action

S-adenosyl methionine (SAMe) is an amino acid found throughout the body, and it acts as a co-substrate for methyl transfer. Methylation is important in many systems, including the methylation of genetic material (an important epigenetic mechanism), and cellular growth and repair. SAMe is synthesised through the pathway known as the one-carbon cycle and is a derivative of folate metabolism, as previously described. Folate and SAMe metabolism are intimately associated through one-carbon metabolism [95]. SAMe is also important in the synthesis of various neurotransmitters in the brain, such as dopamine, serotonin and norepinephrine (also known as noradrenaline) [96].

Studies by Pfeiffer [97] and Edelman [98] demonstrate that SAMe acts as a methyl donor, facilitating the breakdown of histamine into N-methyl-histamine, a non-reactive metabolite. SAMe itself is synthesised from L-methionine and adenosine triphosphate (ATP) via the one-carbon cycle, which requires adequate folate and vitamin B12 levels [99]. This suggests that individuals with high histamine may benefit from SAMe supplementation, potentially normalising brain histamine levels through the process of methylation [97]. Conversely, those with low or normal histamine levels might see less benefit, assuming histamine plays a significant role in their depression. Beyond histamine, Cimino et al. suggest SAMe may influence neuronal function by increasing the conversion of phosphatidylethanolamine to phosphatidylcholine. This enhances cell membrane fluidity, potentially improving neurotransmission through increased receptor availability or efficiency [100].

Membrane dynamics can also significantly impact cellular communication [101,102]. Studies, including that by Cohen et al. (1987) have detailed a decreased membrane microviscosity in patients with dementia treated with intravenous SAMe. The research on depressed outpatients [103], however, has yielded inconsistent results with oral SAMe. While some patients exhibited increased membrane fluidity, others were found to show a decrease or no change.

Another interesting mechanism of action links methylfolate and SAMe metabolism to depression. Homocysteine is normally metabolised to SAMe, with folate as a co-factor. The MTHFR enzyme is also involved in this activity and, as described previously, polymorphisms can lead to reduced enzyme activity. Additionally, a reduction in folate can lead to reduced levels of SAMe which, as explained earlier, reduces the ability to synthesise the neurotransmitters dopamine, serotonin and noradrenalin. Hyperhomocystenaemia can lead to a production of homocysteic acid and cysteine sulfonic acid, which is potentially neurotoxic to dopaminergic nerves [104].

#### 3.3.2. Low SAMe Levels in Depression

While there is not a dietary deficiency of SAMe per se (given it is not available via dietary consumption), SAMe levels in blood have been shown to be significantly lower in women compared to men [105], which is striking, given the higher prevalence of MDD in women [106]. Measurements in cerebral spinal fluid (CSF) performed by Bottilgieri et al. [107] showed low SAMe levels associated with depression. Additionally, the enzyme responsible for SAMe production is lower in patients with depression and schizophrenia [108].

Importantly, oral supplementation of SAMe at a dose level of 1200 mg daily for 4–8 months significantly increased the CSF levels of SAMe, demonstrating that oral intake is able to correct a SAMe deficiency [107]. Similar results were also seen in plasma.

#### 3.3.3. Observational Studies of SAMe in Depression

No observational studies for SAMe have been conducted, as SAMe is not normally obtained through diet; however, a deficiency in SAMe has been linked to depression [108], as a result of low levels of folate and vitamin B12, which are precursors to SAMe [109].

#### 3.3.4. Interventional Studies of SAMe in Depression

Interventional studies with S-adenosylmethionine are presented in Table 5.

Of the 19 interventional studies reported above, 12 showed significant improvements in depression with SAMe supplementation, 2 studies were equivocal (although it should be noted that 1 of the equivocal reports is a sub-analysis from a study without any effect), and 5 studies showed no effect (for some studies potentially due to the high placebo effect).

#### 3.3.5. Guidelines

The World Federation of Societies of Biological Psychiatry (WFSBP) and Canadian Network for Mood and Anxiety Treatments (CANMAT) Taskforce has a “weak recommendation” for 1600 mg–3200 mg of SAMe for adjunctive use in MDD [9]. This position was held based on recent SAMe study data, which did not reveal a statistical significance over a placebo. It is worth noting that there have been concerns raised over some studies having a very high placebo response rate, using too low a dose of SAMe, or stability issues.

## 4. Discussion

The globally increasing prevalence of MDD, combined with the suboptimal response rates to standard antidepressant therapies, creates an urgent need for alternative and/or additional treatment options. Therefore, this article aims to provide a narrative review evaluating the efficacy of three evidence-based nutritional supplements (omega-3 fatty acids, methylfolate, and S-adenosylmethionine (SAMe)) in the management of depression.

A number of notable findings emerged, including (i) omega-3 fatty acids may modulate inflammatory pathways and neurotransmitter functions (which are often dysregulated in MDD), (ii) the role of polymorphisms in the gene for folate metabolism in depression, (iii) methylfolate’s role in the production of neurotransmitters and homocysteine metabolism, and (iv) SAMe’s involvement in certain methylation processes implicated in the onset and treatment of depressive symptoms.

The efficacy of any nutrient for any health claim is dependent on product stability. In the case of omega-3, these lipids are readily oxidised due to their high level of desaturation. Typically, this is reduced by the inclusion of antioxidants in the oil, the use of nitrogen blanketing during the manufacturing of capsules, and the careful monitoring of primary and secondary oxidation products during manufacturing. S-adenosylmethionine is notoriously unstable due to hydrolysis, and commercially this is mitigated by tosylation. However, the compound remains unstable, and studies that do not report stability have the limitation that instability may be a compounding factor.

The complementary but independent biological pathways of the nutrients reviewed also raise a clear possibility of additional benefit when using these nutrients in combination. Whilst combination studies have not been reported, the concept of a multicomponent approach is in keeping with the hypothesis that depression may result from a multiple-pathway aetiology.

The scope of the article has been limited to three nutrients, but this should not be interpreted as support being limited to these three nutrients. Vitamin D is of particular interest, since there is significant interventional data, mechanistic support and positive conclusions from several guidelines. Probiotics is also an area of intense research into the effects of gut health on neurological behaviour with interesting initial results [128], including a potential mechanism for folate action via the gut microbiome [129].

The aim of this paper was to critically review multiple pathways through which three nutrients could impact depression. In order to bring together mechanistic, observational and interventional studies, a narrative review rather than a strict systematic review was deemed most suitable. A systematic review for each nutrient separately is however recognized as providing a more concrete understanding of the role of these nutrients.

A general weakness of nutritional studies is the diversity of study design. Whilst reviews such as this attempt to bring together results from diverse studies, there is an underlying difficulty in summarising the data (albeit qualitatively) from studies with differing design elements. Important design variables include age, sex, ethnicity, inclusion criteria (in the case of depression, the actual form of depression or other mental health diagnosis), background medication, background nutritional status, co-morbidities, physical exercise, and genetic background (including relevant SNPs). This argument, however, can also be turned around, whereby adequate efficacy demonstrated in studies with differing populations and study designs implies a robust effect of nutritional intervention.

Despite these challenges, there is a solid body of work supporting the action of these nutrients, leading to their consideration and inclusion in several guidelines for the management of depression.

## 5. Conclusions

This review identifies a body of evidence supportive of the role of omega-3 fatty acids, methylfolate, and SAMe as useful compounds in the management of depression, while also noting where key research gaps exist. Considerable evidence is presented from epidemiological, mechanistic and interventional studies, supporting their role in depression management. Continuing to investigate the mechanistic pathways and individual factors which determine the effectiveness of these nutraceuticals, either as standalone treatments or in unison with each other and other pharmaceutical options, will prove valuable for informing the clinical and personal use of nutrition for managing depression.

## Figures and Tables

**Table 1:** Epidemiological studies of seafood/omega-3 and depression.

Study	Design	Outcome
Bidirectional longitudinal associations of omega-3 polyunsaturated fatty acid plasma levels with depressive disorders [19]	Baseline and follow-up data collected from 2912 subjects with depressive disorder. Omega-3 levels measured by nuclear magnetic resonance.	n-3PUFA levels were significantly reduced at baseline in depressives compared to healthy participants. Changes in depressive disorders was not associated with a change in omega-3 levels.
Dietary intake of fish and *n*-3 polyunsaturated fatty acids and risk of postpartum depression: a nationwide longitudinal study—the Japan Environment and Children’s Study (JECS) [20]	A total of 81,294 women were followed up after 1 year post birth. Omega-3 intake was evaluated using a food frequency questionnaire, and depression rated using the Kessler Psychological Distress Scale.	A reduced risk of postpartum depression at was seen at 6 months when comparing omega-3/fish intake from the second to fifth quintiles vs. the lowest quintile. At 1 year follow-up, mental illness was reduced when comparing quintiles
Serum *n*-3 polyunsaturated fatty acids are inversely associated with longitudinal changes in depressive symptoms during pregnancy [21]	Cohort of 172 Brazilian women. Depression was measured with the Edinburgh Postnatal Depression Scale during pregnancy. Fatty acid measurements were taken from blood samples.	Higher concentrations of EPA were associated with lower odds of depressive symptoms (OR 0.92). Depressive symptoms were decreased with increasing concentrations of total omega-3 fatty acids, α-linolenic acid, DPA, and DHA.
The Omega-3 Index Is Inversely Associated with Depressive Symptoms among Individuals with Elevated Oxidative Stress Biomarkers [22]	A total of 787 US based Puerto Ricans enrolled. Oxidative stress, omega-3 index and fatty acid profile, and depression rates (CES-D) were assessed for 2 years.	Amongst subjects with elevated oxidative stress, depressive symptoms decreased with increasing omega-3 index. This suggests that oxidative stress may identify individuals that would benefit from omega-3 consumption.
High Levels of Depressive Symptoms in Pregnancy With Low Omega-3 Fatty Acid Intake From Fish [23]	Cohort study of pregnant women in the UK with an initial size of 14,541 pregnant women. Edinburgh Postnatal Depression Scale (EPDS) was used to measure at 32 weeks gestation.	Women consuming more than 1.5 g of omega-3 fatty acids per week were more likely to have reduced levels of depression compared to those consuming no omega-3 fatty acids.
Fish and *n*-3 polyunsaturated fatty acid intake and depressive symptoms [24]	Cross-sectional study in Japanese high schools with 3067 boys and 3450 girls aged 12–15. Self-administered dietary questionnaire and depression measured with Epidemiologic Studies Depression scale score ≥16.	In male subjects, a lower rate of depression was noted in those with a higher intake of fish, EPA and DHA. No effect was seen in female subjects.
Fatty acids intake and depressive symptomatology in a Greek sample: an epidemiological analysis [25]	A total of 453 men and 400 women aged 18–65 were assessed for depression using the Zung´s self-rating depression scale. A food questionnaire was used to estimate omega-3 dietary intake and omega-3 fatty acids measured in plasma.	Increased plasma levels of polyunsaturated, in particular EPA and DHA, as well as monounsaturated fatty acids, were associated with lower levels of depression.
Higher dietary intake of long-chain omega-3 polyunsaturated fatty acids is inversely associated with depressive symptoms in women [26]	Cohort study of 3317 from a Coronary Artery Risk Development study. Dietary intake of EPA, DHA or a combination was assessed.	The highest intake quintiles were associated with low risk of depressive symptoms. Associations were pronounced for women.
Depressive symptoms during pregnancy in relation to fish consumption and intake of *n*-3 polyunsaturated fatty acids [27]	Cohort study of 2394 pregnant women assessing pre-natal depression and dietary intake of EPA and DHA. Depressive symptoms were measured using the Center for Epidemiologic Studies—Depression Scale (CES-D). Intakes of fish and EPA+DHA were measured using a validated food-frequency questionnaire.	Depression was not associated with dietary intake of EPA and DHA, except in certain sub-populations (smokers and single mothers).
Dietary intake of folate, other B vitamins, and omega-3 polyunsaturated fatty acids in relation to depressive symptoms in Japanese adults [28]	A total of 309 men and 218 women aged 21 to 67 years of age were assessed for depression using the Center for Epidemiologic Studies Depression scale and dietary intake was assessed by questionnaire.	There was no association observed between depression and dietary intake.
Fish consumption and risk of depression: Epidemiological evidence from prospective studies [29]	A meta-analysis of 10 cohort studies with 6672 cases of depression from 109,764 subjects.	A modest inverse association between fish or omega-3 fatty acid intake and risk of depression, especially in women.
Polyunsaturated Fatty Acids in Perinatal Depression: A Systematic Review and Meta-analysis [30]	Assessed levels of PUFA in pre-natally depressed women in 12 studies.	Significantly lower levels of EPA and DHA and increased omega-6:omega-3 ratios were observed in pre-natal depressed women.
Fish consumption and depression: the Northern Finland 1966 birth cohort study [31]	A total of 5689 men and women were followed from birth to 30. Amongst other things, fish consumption and depression were monitored.	In women, the risk of developing depression increased with low fish consumption but no association was seen in men.

**Table 2:** Interventional studies of omega-3 in depression (post-2016).

Study	Design	Outcome
Clinical response to EPA supplementation in patients with major depressive disorder is associated with higher plasma concentrations of pro-resolving lipid mediators [16,35]	A total of 61 patients with MDD enrolled to 1 of 4 arms, EPA at 1, 2 or 4 g/day or placebo. Depression was assessed using ICS-C30 scale.	Response rates greater in those taking 4 g EPA/day and those with highest resolvin levels. These also showed the greatest reduction in CRP.
Omega-3 polyunsaturated fatty acids in cardiovascular diseases comorbid major depressive disorder—Results from a randomized controlled trial [36]	A total of 59 patients with CVD and MDD randomised to take 2 g EPA + 1 g DHA per day or placebo for 12 weeks.	No overall effect of *n*-3 PUFA, but improvement of core depression symptoms was seen in the very severe MDD group with *n*-3 PUFA.
Plasma estradiol levels and antidepressant effects of omega-3 fatty acids in pregnant women [37]	A total of 108 pregnant women with Edinburgh Postnatal Depression Scale scores ≥9 measured at 12–24 weeks gestation. Subjects took 1206 mg EPA or placebo daily for 12 weeks.	Increase in EPA was significantly associated with a decrease in depressive symptoms. There were no EPA associated changes in inflammatory cytokines.
Omega-3 polyunsaturated fatty acids and psychological intervention for workers with mild to moderate depression: A double-blind randomized controlled trial [38]	Subjects took 1 g EPA and 0.5 g DHA daily for 12 weeks or placebo.	The study suffered from a high number of drop-outs, reducing the power of the study. The omega-3 and placebo group showed improved depression scores, but no significant difference was seen between the groups.
A Mediterranean-style dietary intervention supplemented with fish oil improves diet quality and mental health in people with depression: A randomized controlled trial (HELFIMED) [39]	A total of 95 subjects suffering with depression were assessed for quality of life at 3 and 6 months, following different dietary plans including fish oil supplementation. Red blood cell omega-3 levels were measured.	Omega-3 levels were associated with improved depression. Healthy dietary changes are achievable and, supplemented with fish oil, can improve mental health in people with depression.
Pilot Randomized Controlled Trial of Omega-3 and Individual-Family Psychoeducational Psychotherapy for Children and Adolescents With Depression [40]	A total of 72 youths randomised to omega-3 (700 mg EPA and 100 mg DHA daily) or placebo for 12 weeks.	Omega-3 was well tolerated and gave better remission rates than psychoeducational therapy alone, and placebo.
Influence of adjuvant omega-3-polyunsaturated fatty acids on depression, sleep, and emotion regulation among outpatients with major depressive disorders—Results from a double-blind, randomized and placebo-controlled clinical trial [41]	A total of 50 outpatients with MDD were randomised to omega-3 or placebo for 12 weeks. Questionnaires for depression, anxiety, sleep, intolerance and emotional regulation were taken and experts made a blinded assessment using the Montgomery-Asberg Depression scale.	Depression by self-assessment and expert opinion decreased in the O3 group to a greater extent than placebo. PUFA gave a greater reduction in anxiety, an intolerance of uncertainty, and sleep disturbance.
Long-Chain Omega-3 Fatty Acid Supplements in Depressed Heart Failure Patients: Results of the OCEAN Trial [42]	A total of 108 coronary heart failure patients with MDD. A 3-way randomization, 2 g of 400:200 or pure EPA or placebo for 12 weeks.	Omega-3 content in red blood cells increased in those taking O3 supplementation with associated improvements in cognitive depressive symptoms (improvement was related to dose), social function.
A Double-Blind Placebo-Controlled Trial of Omega-3 Fatty Acids as a Monotherapy for Adolescent Depression [43]	A total of 51 adolescents with MDD diagnosis randomised to omega-3 (2:1 ratio of EPA:DHA with a start dose of 1.2 g daily and increasing to 3.6 g daily) or placebo for 10 weeks with increasing doses every 2 weeks.	Omega-3 supplementation provided no benefit compared to placebo on any clinical feature, including depression severity.
The influences of vitamin D and omega-3 co-supplementation on clinical, metabolic and genetic parameters in women with polycystic ovary syndrome [44]	A total of 60 women with polycystic ovary syndrome randomised to vitamin D (50,000 IU) plus omega-3 (2 g daily) or placebo for 12 weeks.	Omega-3 and vitamin D resulted in significant improvements in Beck Depression Inventory, anxiety and stress scale scores.
Omega-3 supplementation effects on body weight and depression among dieter women with co-morbidity of depression and obesity compared with the placebo: A randomized clinical trial [45]	A total of 65 overweight/obese with depression randomised to 6 capsules of omega-3 (180 mgEPA and 120 mg DHA per cap) or placebo for 12 weeks.	Omega-3 significantly reduced depression compared to placebo. Weight was also significantly reduced in the omega-3 group.
Omega-3 fatty acids for a better mental state in working populations—Happy Nurse Project: A 52-week randomized controlled trial [46]	A total of 80 nurses were randomised to omega-3 (1200 mg EPA, 600 mg DHA, daily) or placebo for 13 weeks.	No effect of omega-3 was seen; however, depression scores were measured at weeks 26.
The effects of omega-3 and vitamin E co-supplementation on parameters of mental health and gene expression related to insulin and inflammation in subjects with polycystic ovary syndrome [47]	A total of 40 women with polycystic ovary syndrome were randomised to 1 g omega-3 plus vitamin E or placebo for 12 weeks.	Omega-3/vitamin E compared with placebo, led to significant improvements in stress score scales, Beck depression inventory total score, and depression anxiety.
Omega-3 supplementation associated with improved parent-rated executive function in youth with mood disorders: secondary analyses of the omega-3 and therapy (OATS) trials [48]	A total of 95 youths with depression or bipolar disorder were randomised to 2 capsules twice daily of omega-3 (reported as 1.87 g mostly EPA) or placebo for 12 weeks.	Omega-3 supplementation, as either monotherapy or adjunctive (aggregated), showed significant improvement compared to placebo.
Adjunctive low-dose docosahexaenoic acid (DHA) for major depression: An open-label pilot trial [49]	A total of 28 patients with MDD in an open label study taking low-dose DHA (260 mg or 520 mg/day) for 8 weeks.	After 8 weeks there was a >50% reduction in the HAM-D scale. the results suggest that DHA may provide additional adjunctive benefits in patients with mild- to -moderate depression.
Emulsified omega-3 fatty-acids modulate the symptoms of depressive disorder in children and adolescents: a pilot study [50]	A total of 38 children with depressive disorder were randomised to omega-3 (1 g EPA and 750 mg DHA daily) or omega-6 comparator. Childrens’ depression inventory ratings were performed every 2 weeks during 12 weeks of intervention.	Significant reduction in depression (CDI) scores were seen after 12 weeks intervention with the omega-3 group. A greater improvement was seen with those with depressive disorder compared to mixed anxiety depressive disorder.
Omega-3 supplementation from pregnancy to postpartum to prevent depressive symptoms: a randomized placebo-controlled trial [51]	A total of 60 women at risk for postpartum depression were randomised to fish oil (1.08 g EPA, 0.72 g DHA) or placebo for 16 weeks. The Edinburgh Postnatal Depression Scale (EPDS) was used, and serum fatty acids measured for compliance.	Increases in plasma EPA and DHA were seen with the fish oil group. No difference between the intervention and control groups was seen for EPDS > 11, EPDS over time or from pregnancy to postpartum.
Lipid correlates of antidepressant response to omega-3 polyunsaturated fatty acid supplementation: A pilot study [52]	A total of 16 patients with major depressive disorder were given fish oil (1.6 g EPA and 0.8 g DHA daily) for 6 weeks. Depression was rated using the Hamilton Depression Rating Scale.	A reduction in depression severity showed a relationship with DHA plasma phospholipids. 5 patients showed remission with higher levels of DHA compared with non-responders.
Oxidative stress predicts depressive symptom changes with omega-3 fatty acid treatment in coronary artery disease patients [53]	A total of 79 patients with coronary artery disease were recruited with depression measured using the 17-item Hamilton Depression Rating Scale. Patients were randomised to 1.9 g PUFA or placebo for 12 weeks.	The *n*-3 PUFA group was associated with greater depressive symptom improvement. No association was found with the placebo group.
Psychoeducational Psychotherapy and Omega-3 Supplementation Improve Co-Occurring Behavioral Problems in Youth with Depression: Results from a Pilot RCT [54]	A total of 72 youths with depression were randomised to one of four treatments for therapy (PEP) and omega-3 (1.4 g EPA and 200 mg DHA daily). Treatments were PEP+omega3, PEP monotherapy, omega-3 monotherapy or placebo. Assessments were made every 2 weeks up to 12 weeks. Assessments were made using the Eyberg Child Behaviour Inventory.	Youths receiving combined treatment had a significant behavioural improvement.
Beneficial effects of dietary docosahexaenoic acid intervention on cognitive function and mental health of the oldest elderly in Japanese care facilities and nursing homes [55]	A total of 75 participants with dementia were randomised to receive meals containing 1720 mg DHA or a placebo for 12 months. Assessments were made using the Mini-Mental State Examination and Hasegawa’s Dementia Scale—Revised. The subjects mental health and self-rated depression were also measured.	The DHA group showed a tendency for greater mental health, less apathy, and reduced depression compared to the placebo group. DHA supplementation protects against age-related decline.

**Table 3:** Observational studies investigating folate deficiency in psychiatric disorders.

Study	Design	Outcome
Serum and red blood cell folate in depression [73]	Case–control; 95 with major depression, 60 controls.	Significantly reduced serum and red blood cell folate levels in MDD vs. controls. Lower serum levels were associated with severity of depression.
Red cell folate concentrations in psychiatric patients [74]	Case–control; 152 psychiatrist-diagnosed depressed, 42 controls	Depressed patients had significantly reduced red cell folate levels vs. euthymic patients.
A controlled study of folate levels in Chinese inpatients with major depression in Hong Kong [75]	Case–control; 117 major depressed, 72 without history of depression	Significantly lower mean serum levels of folate were seen in patients vs. control subjects.
Vitamin B12, folate, and homocysteine in depression: the Rotterdam Study [76]	Patients > 55 years of age. DSM-IV depression or dysthymia, *n* = 112; 416 non-depressed age-matched	Folate, vitamin B12, and hyperhomocysteinaemia were associated with depression.
Vitamin B (12) deficiency and depression in physically disabled older women: epidemiologic evidence from the Women’s Health and Aging Study [77]	Women > 65 years of age; 122 with severe depression on GDS score > 14, 478 controls with GDS < 9	No serum folate deficiency seen. Significant vitamin B12 deficiency in depressed subjects related to severity.
Folate, vitamin B12, homocysteine, and the MTHFR 677C->T polymorphism in anxiety and depression: the Hordaland Homocysteine Study [69]	Patients 46–49 and 70–74; 243 with HAD > 8, 5705 non depressed controls.	Hyperhomocystenaemia was not related to plasma levels of folate or vitamin B12 but showed significant association with depression.
Dietary folate and depressive symptoms are associated in middle-aged Finnish men [78]	Population between 42 and 60 years of age; 2682 men and women recruited, 228 had elevated depression.	Subjects in the lowest third of folate intake showed significantly higher risk of having depressive symptoms
Dietary folate and the risk of depression in Finnish middle-aged men [79]	Population cohort of men 42–60 years old; 47 with diagnosis of major depression and 2313 controls without diagnosis of depression.	Low dietary intake of folate is a risk factor for severe depression.
Depression and folate status in the US Population [80]	Population cohort of subjects 15–39 years of age; 301 with major depression (DSM-III), 121 with dysthymia and 2526 controls.	Folate concentrations in serum and red blood cells were significantly lower in depressed subjects than those who had never been depressed. Dysthymic subjects had lower RBC folate but not serum folate compared to those who had never been depressed.
Plasma folate concentrations are associated with depressive symptoms in elderly Latina women despite folic acid fortification [81]	Latino population ≥ 60 years of age; 385 depressed with CES-D score > 15, 1125 controls with CES-D ≤ 15.	Low folate status was associated with depressive symptoms in Latino women but not men.
Dietary intake of folate, other B vitamins, and omega-3 polyunsaturated fatty acids in relation to depressive symptoms in Japanese adults [28]	A total of 309 men and 218 women aged 21 to 67 years of age were assessed for depression using the Center for Epidemiologic Studies Depression scale and dietary intake assessed by questionnaire.	Higher dietary intake of folate was associated with a lower prevalence of depressive symptoms in Japanese men but not women.

**Table 4:** Intervention studies with folate/methylfolate in MDD.

Study	Design	Outcome
L-methylfolate Augmentation to Antidepressants for Adolescents with Treatment-Resistant Depression: A Case Series [84]	A total of 10 females with treatment resistant depression, with 80% showing SNPs in MTHFR gene.	A total of 80% showed improvement in depression, anxiety and irritability with L-methylfolate.
L-methylfolate as adjunctive therapy for SSRI-resistant major depression: results of two randomized, double-blind, parallel-sequential trials [85]	A total of 148 outpatients with SSRI resistant MDD were randomised to arms with L-methylfolate (7.5 mg daily) or placebo. A second study included 75 patients with the same protocol but higher doses of L-methylfolate (15 mg daily).	The first arm with 7.5 mg/day L-methyl folate showed no effect on outcome. The second trial showed a significant effect of intervention. No safety findings.
L-methylfolate Plus SSRI or SNRI from Treatment Initiation Compared to SSRI or SNRI Monotherapy in a Major Depressive Episode [86]	A retrospective analysis of L-methylfolate (7.5 or 15 mg) plus SSRI/SNRI in 242 subjects after 60 days therapy. Depression was measured with CGI-S.	L-methylfolate plus antidepressant treatment was more effective than monotherapy in depression, and the numbers showed major improvement and reduced time until improvement.
Effect of adjunctive L-methylfolate 15 mg among inadequate responders to SSRIs in depressed patients who were stratified by biomarker levels and genotype: results from a randomized clinical trial. [87]	A total of 75 patients with SSRI-resistant MMD received 15 mg/d methylfolate for 60 days or placebo for 30 days followed by methylfolate for 30 days, or 60 days of placebo.	Significant improvements in depression scores were seen in populations selected by specific depression biomarkers
L-Methylfolate for Bipolar I depressive episodes: An open trial proof-of-concept registry [88]	Open label, single group study of 10 subjects with bipolar depression treated with 15 mg methylfolate and concurrent treatment for 6 weeks.	L-methylfolate in combination with treatment, as usual, has potential to treat bipolar depression.
Long-term efficacy, safety, and tolerability of L-methylfolate calcium 15 mg as adjunctive therapy with selective serotonin reuptake inhibitors: a 12-month, open-label study following a placebo-controlled acute study [89]	Analysis from 2 separate studies. Treatment-resistant depression with 15 mg methylfolate for 12 months. Of the 68 subjects in the open-label phase, 38% (*n* = 26) showed full recovery, without any recurrence of MDD.	Adjunctive L-methylfolate 15 mg/d may be suitable for patients who fail to adequately respond to antidepressant monotherapy, with preliminary evidence demonstrating sustained remission and sustained recovery.
Enhancement of recovery from psychiatric illness by methylfolate [90]	The study measured the folate level in blood and effect of supplementation of 15 mg methylfolate for 6 months.	A total of 33% of subjects had folate deficiency. Methylfolate significantly improved clinical endpoints in depressive patients.
Folinic acid (Leucovorin) as an adjunctive treatment for SSRI-refractory depression [91]	A total of 22 adults with MDD and partial or nonresponsive to SSRI after at least 4 weeks of treatment. Open label single arm trial with folinic acid (which is metabolised to methylfolate).	Folate levels rose in treated subjects. However, only 31% completed and 27% achieved response, and only 19% completed and 18% achieved remission. Leucovorin appears to be modestly effective.
A prenatal supplement with methylfolate for the treatment and prevention of depression in women trying to conceive and during pregnancy [92]	A non-blinded, 12-week study in women with histories of MDD planning pregnancy or who were pregnant < 28 weeks. Group 1 were healthy controls (not depressed). Group 2 participants were women with MDD.	Group 2 (*n*= 6) showed significant improvements in depression (MADRS) scores (*p* = 0.001), with 5 participants improving >50% and 1 participant improving.
Assessing Effects of l-Methylfolate in Depression Management: Results of a Real-World Patient Experience Trial [93]	A total of 502 patients with MDD received 15 mg or 7.5 mg methylfolate for 3 months, without an anti-depressant. Depression was measured with the PHQ-score.	Use of L-methylfolate achieved statistically significant improvements in self-reported depression symptoms.
A retrospective examination of adjunctive L-methylfolate in children and adolescents with unipolar depression [94]	A total of 412 patients were retrospectively assessed for MTHFR genotype. Patients were more likely prescribed methylfolate if they carried a relevant SNP.	No difference was seen between methylfolate treated or non-treated groups.
L-methylfolate Plus SSRI or SNRI from Treatment Initiation Compared to SSRI or SNRI Monotherapy in a Major Depressive Episode [86]	The study was a retrospective study of subjects with MDD receiving SSRI/SNRI at treatment initiation with (*n* = 95) or without (*n* = 147) methylfolate. Depression was measured using CGI-S scores at 60 days.	Major improvement experienced by 18.5% of methylfolate patients compared to 7.04% with only SSRI/SNRI. The combination of L-methylfolate with SSRI/SNRI at treatment onset was more effective in improving depressive symptoms than antidepressant monotherapy

**Table 5:** Nutritional studies with SAMe supplementation.

Study	Design	Outcome
A Prospective Randomized Double-Blind Study Evaluating UP165 and S-Adenosyl-l-Methionine on Depression, Anxiety and Psychological Well-Being [110]	42 subjects were randomised to 8 weeks of supplementation with corn or SAMe (400 mg/day). Questionnaires performed at baseline, 4 and 8 weeks. The primary endpoint was upon seeing the effect of corn leaf derivative on depression. The secondary endpoint was upon seeing the effect of SAMe on depression.	SAMe demonstrated a trend for improvement.
Dose increase of S-Adenosyl-Methionine and escitalopram in a randomized clinical trial for major depressive disorder [111]	Subjects who showed no effect of 1600 mg SAMe recruited to test effect of 3200 mg SAMe.	Dose effect was seen at 3200 mg.
S-adenosyl-L-methionine (SAMe) as an adjunct for resistant major depressive disorder: an open trial following partial or nonresponse to selective serotonin reuptake inhibitors or venlafaxine [112]	Thirty anti-depressant-treated subjects with chronic MDD received 800 to 1600 mg of tosylated SAMe for 6-weeks.	Supplementation with SAMe led to a response rate of 50% and a remission rate of 43%.
S-Adenosyl-L-Methionine augmentation in patients with stage II treatment-resistant major depressive disorder: an open label, fixed dose, single-blind study [113]	Thirty-three outpatients with MDD who were resistant to at least 8 weeks of treatment with antidepressants received SAMe (800 mg) for 8 weeks, as an adjunct to existing medication.	A significant decrease in depression (HAM-D score) was observed with SAMe supplementation. A response was seen in 60% of patients and remission in 36%.
Efficacy and tolerability of oral and intramuscular S-adenosyl-L-methionine 1,4-butanedisulfonate (SAMe) in the treatment of major depression: comparison with imipramine in 2 multi-center studies [114]	A total of 143 MDD subjects received oral SAMe 1600 mg daily for 6 weeks vs. 138 subjects on imipramine in a second arm. Imipramine is a tricyclic antidepressant.	The antidepressive efficacy of 1600 mg SAMe/d orally and 400 mg SAMe/d intramuscularly is comparable with that of 150 mg imipramine/d orally, but SAMe is significantly better tolerated.
S-Adenosylmethionine (SAMe) monotherapy for depression: an 8-week double-blind, randomised, controlled trial [115]	A double-blinded 8-week, randomised study in 49 patients with MDD. Subjects received either 800 mg/day of SAMe tosylate monotherapy or placebo.	The study demonstrated a clinically relevant reduction in depression from baseline to week 8 in favour of SAMe over placebo. Despite this, no significant effect was seen, authors noting due potentially to a high placebo effect.
S-adenosyl methionine (SAMe) versus escitalopram and placebo in major depression RCT: efficacy and effects of histamine and carnitine as moderators of response [116]	A total of 144 subjects with MDD randomised to SAMe (1600–3200 mg/daily), escitalopram (10–20 mg/daily), or matching placebo for 12 weeks of double-blind treatment. Data analysis was from a larger study and involved more men than women. Depression was measured using HAMD-17.	In this sub-analysis study, a significant improvement was seen between groups after 12 weeks of supplementation.
Bioavailability of S-adenosyl methionine and impact on response in a randomized, double-blind, placebo-controlled trial in major depressive disorder [117]	A total of 35 subjects received either placebo or 800–1600 mg/day SAMe for a duration of 6 weeks in partial SSRI responders with MDD.	Blood levels of SAMe were increased with supplementation. No clinical effect was seen.
S-adenosyl methionine (SAMe) augmentation of serotonin reuptake inhibitors for antidepressant non-responders with major depressive disorder: a double-blind, randomized clinical trial [118]	A total of 73 serotonin reuptake inhibitor (SRI) non-responders with major depressive disorder enrolled in a 6-week, double-blind, randomised trial of adjunctive oral SAMe (target dose: 800 mg/twice daily). Subjects continued on the standard treatment. The primary outcome measure for the study was the response rates according to the 17-item Hamilton Depression Rating Scale (HAM-D).	Subjects receiving SAMe supplementation showed higher HAM-D response (36%) and remission rates (26%) compared to placebo (17.6% HAM-D and 11.7% remission).
Oral Administration of S-Adenosylmethionine (SAMe) and *Lactobacillus Plantarum* HEAL9 Improves the Mild-To-Moderate Symptoms of Depression: A Randomized, Double-Blind, Placebo-Controlled Study [119]	A total of 90 patients were randomised to receive either SAMe (200 mg daily) plus L. plantarum HEAL9 (*n* = 46) or placebo (*n* = 44) groups for 6 weeks. Depression was measured using the Z-SDS score as the primary endpoint.	The combination product provided a greater reduction in depression score compared to placebo (*p* = 0.0165).
An augmentation study of MSI-195 (S-adenosylmethionine) in Major Depressive Disorder [120]	A 6-week double-blind, placebo-controlled augmentation study (800 mg) comparing S-adenosylmethionine or placebo added to antidepressant medication in acutely depressed subjects with Major Depressive Disorder. Scales used were (HamD17, MADRS, IDS-SR30)	There were no overall statistically significant differences found between groups.
Adjunctive S-adenosylmethionine (SAMe) in treating non-remittent major depressive disorder: An 8-week double-blind, randomized, controlled trial [121]	An 8-week, double-blind RCT, in which 107 treatment non-remittent outpatients with DSM-5 diagnosed MDD were randomised to either SAMe tosylate (800 mg) or placebo adjunctively to antidepressants.	No significant between-group difference observed. Due to such a distinctly high placebo-response, Author’s commented that future studies should employ a placebo run-in period.
Nutraceuticals for major depressive disorder- more is not merrier: An 8-week double-blind, randomised, controlled trial [122]	158 outpatients with MDD were randomised to receive a combination of either: SAMe (800 mg); Folinic acid; Omega-3 fatty acids; 5-HTP, Zinc picolinate, and relevant co-factors or placebo. The primary outcome was change in MADRS score.	The placebo showed a higher response rate than the nutrient combination.
Is S-Adenosyl Methionine (SAMe) for Depression Only Effective in Males? A Re-Analysis of Data from a Randomized Clinical Trial [123]	A total of 189 subjects were recruited to a double-blind RCT, where subjects received either 1600–3200 mg/day SAMe or placebo or a comparator SSRI. This study is a post hoc sub-analysis of Mischoulon et al. reported below [124].	In the main study, SAMe did not show any effect on depression. In this sub-analysis, SAMe was superior to placebo only in males.
Effects of S-adenosylmethionine augmentation of serotonin-reuptake inhibitor antidepressants on cognitive symptoms of major depressive disorder [125]	A total of 46 subjects with MDD, non-responsive to SSRIs, were randomised to SAMe or placebo for 6 weeks. Efficacy was assessed with the self-rated cognitive and physical symptoms questionnaire (CPFQ), used to determine MDD and symptoms before and after treatment.	Those receiving SAMe showed a greater improvement in the ability to recall information (*p* = 0.04). There was also a trend.
A double-blind, randomized, placebo-controlled clinical trial of S-adenosyl-L-methionine (SAMe) versus escitalopram in major depressive disorder [124]	One hundred eighty-nine outpatients with MDD defined by DSM-IV were recruited. Subjects were randomised to either SAMe (1600–3200 mg), escitalopram (10–20 mg) or placebo for 13–18 weeks.	Although remission rates for SAMe (28%) were equal, there was no statistical improvement compared to placebo (17% remission rate).
A double-blind, randomized parallel-group, efficacy and safety study of intramuscular S-adenosyl-L-methionine 1,4-butanedisulphonate (SAMe) versus imipramine in patients with major depressive disorder [126]	Subjects were randomised to receive either SAMe at 400 mg/day (*n* = 146) and or an anti-depressant (IMI, *n* = 147) for 4 weeks. The primary efficacy measurements were HAMD score and the Clinical Global Impression (CGI) at week 4. SAMe was injected intra-muscularly.	The study showed an equal response between the nutrient SAMe and the anti-depressant, but SAMe was significantly better tolerated.
Double-blind, placebo-controlled study of S-adenosyl-L-methionine in depressed postmenopausal women [127]	A total of 80 women, between the ages of 45 and 59, who were diagnosed as having DSM-III-R major depressive disorder or dysthymia between 6 and 36 months following either natural menopause or hysterectomy, underwent 1 week of single-blind placebo washout, followed by 30 days of double-blind treatment with either SAMe 1600 mg/day or placebo.	There was a significantly greater improvement in depressive symptoms in the group treated with SAMe vs. the placebo group from day 10 of the study.
The antidepressant potential of oral S-adenosyl-l-methionine [103]	A total of 20 outpatients were recruited to this open label study. All subjects had MDD with a history of anti-depressant use; 99 subjects had a history or response to treatment and 11 with a prior history of antidepressant nonresponse.	Supplementation with SAMe resulted in an improvement in depression, with an improvement in 7 of 11 treatment-responsive and 2 of 9 treatment-resistant patients experiencing a full response.
